# Effectiveness of a school-based physical activity intervention on obesity in school children: a nonrandomized controlled trial

**DOI:** 10.1186/1471-2458-14-1282

**Published:** 2014-12-16

**Authors:** Xiao-Hui Li, Shenting Lin, Hongxia Guo, Yanli Huang, Lijing Wu, Zilong Zhang, Jun Ma, Hai-Jun Wang

**Affiliations:** Institute of Child and Adolescent Health, School of Public Health, Peking University, No. 38 Xueyuan Road, Haidian District, Beijing, 100-191 China; Department of School Health, Center for Disease Control and Prevention of Changping District, Beijing, 102200 China

## Abstract

**Background:**

Childhood obesity has been a serious public health problem. An effective school-based physical activity (PA) intervention is still lacking in China. This study aimed to assess the effectiveness of a school-based physical activity intervention during 12 weeks on obesity and related health outcomes in school children.

**Methods:**

It was a non-randomized controlled trial. Altogether 921 children aged 7 to 15 years were recruited at baseline survey. Children in the intervention group (n = 388) participated in a multi-component physical activity intervention during 12 weeks that included improvement of physical education, extracurricular physical activities for overweight/obese students, physical activities at home, and health education lectures for students and parents. Children (n = 533) in the control group participated in usual practice.

**Results:**

Participants had mean age of 10.4 years, mean body mass index (BMI) of 19.59 kg/m^2^, and 36.8 % of them were overweight or obese at baseline survey. The change in BMI in intervention group (−0.02 ± 0.06 kg/m^2^) was significantly different from that in control group (0.41 ± 0.08 kg/m^2^). The adjusted mean difference was −0.43 kg/m^2^ (95% CI: −0.63 to −0.23 kg/m^2^, *P* < 0.001). The effects on triceps, subscapular, abdominal skinfold thickness and fasting glucose were also significant in intervention group compared with control group (all *P* < 0.05). The change in duration of moderate to vigorous physical activity (MVPA) in intervention group (8.9 ± 4.3 min/day) was significantly different from that in control group (−13.8 ± 3.3 min/day). The adjusted mean difference was 22.7 min/day (95% CI: 12.2 to 33.2 min/day, *P* < 0.001).

**Conclusions:**

The school-based, multi-component physical activity intervention was effective to decreasing levels of BMI, skinfold thickness, fasting glucose and increasing duration of MVPA. These findings provided evidence for the development of effective and feasible school-based obesity interventions.

**Trial registration:**

Clinicaltrials.gov Identifier: NCT02074332 (2014-02-26)

## Background

Childhood obesity has been a serious public health problem both in developed and developing countries [1–3], which is the major public health challenge of the 21st century [4, 5]. The World Health Organization (WHO) estimates that approximately 155 million school-age children (5–17 years old) were overweight or obese in the world in 2009, and one in every ten school-age children was overweight or obese [6]. The prevalence of overweight and obesity in Chinese 7 to 18-year-old children has continuously increasing from 1985 to 2010 [7]. Childhood overweight and obesity are associated with high risk of adulthood overweight or obesity [8], a series of health problems, such as insulin resistance, hypertension, hyperlipidemia, type 2 diabetes, sleep apnea, gallbladder disease [9, 10], and psychosocial problems [11]. Thus, prevention and control for childhood overweight and obesity are urgently needed.

The cause of obesity is multi-factorial, long-term imbalance between energy intake and energy expenditure (basal/resting, physical activity (PA) and thermogenic response to feeding) [12]. PA accounts for 25%-35% of total energy expenditure in children [13] and has been regarded as a key component in obesity prevention [14].

However, with social development and changes in people’s lifestyles, currently children and adolescents are generally lack of PA. Results from the 2011 national Youth Risk Behavior Surveillance (YRBS) indicated that only 28.7% of nationwide students in the United States were physically active for a total of least 60 minutes per day on each of the 7 days before the survey [15]. Meanwhile, physical inactivity is also increasing in developing countries [16, 17]. In China, the 2010 National Physical Fitness and Health Surveillance reported that a total of 22.7% students aged 9–18 were physically active doing any kind of PA for a total of 60 minutes or more per day [18]. Therefore, it is of vital importance to explore effective and feasible interventional strategies to promote childhood PA, which is helpful for obesity prevention and control.

The school setting is an attractive point for childhood obesity prevention and treatment, where decisions concerning PA, food choices, attendance can be reasonably controlled and programmatically altered [19], and adherence with interventions can be improved [20]. The WHO specifically identified schools as a target setting for the promotion of PA among children and youth [21]. The effectiveness of PA intervention is still inconsistent. Despite the positive effects in a few studies targeting school-based PA [22, 23], there were also disappointing results [20]. Although nutrition component was adopted in some previous obesity interventions, current evidence didn’t show greater effect of PA plus nutrition compared with PA used in isolation [24, 25]. Besides, in most Chinese school-based obesity intervention programs [26–28], PA component was adopted as main strategy, while nutrition component acted as subsidiary part mostly. This is due to lack of cafeteria in many Chinese elementary and middle schools, and diversification and complexity of Chinese food, making it not feasible to adopt nutrition intervention and calculate nutrition intake in Chinese school settings. Thus, we did not include nutrition component in our intervention. Instead, we included some components of healthy eating in health education lectures.

Current research suggested that school-based PA intervention on obesity should include some focus on physical education (PE), involve curriculum on healthy lifestyles, more sessions for PA, create supporting environment and engage with parents [24, 29]. In China, the previous intervention studies regarding PA were very simple, which mainly focused on health education and additional in-school PA [26, 27]. An effective multi-component school-based PA intervention in children is still lacking.

### Theoretical framework

Social ecological model of health promotion has been increasingly used [30, 31], as simple interventions are not likely to work on their own and the effective intervention programs require strategies that affect multiple settings simultaneously [32]. This intervention study is based on the social ecological model [31] that includes concentric rings that influence lifestyle patterns. The "psychobiologic core (individual child)" of the model refers to the genetic, physiologic, and socio-cultural forces that shape one’s identity. The individual child is surrounded by the microsystem, the immediate environments with which a child interacts (his or her parents, siblings, teachers, peers, etc.). The exosystem includes environments with which the child doesn't usually directly interact, but can still affect the child (school boards, parents’ workplace, etc.). The macrosystem includes the broad societal settings under which the others function (shared culture, history, social norms, system of laws, economic system, etc.).

Since children spend most of their time at school and at home, and school setting has attractive points in PA promotion, these settings were targeted (school primarily and family secondarily) in the intervention. At individual level, we enhanced students’ PA duration and intensity, and provided knowledge through health education and materials. At school setting, we improved PE, created more exercise opportunities and enriched health education content. At family setting, we provided family PA guidance and promoted parents’ encouraging and supervising function.

This study applied a non-randomized controlled trail, aiming to identify the effectiveness of a school-based multi-component PA intervention. For assessing effectiveness, we analyzed the data from the baseline survey and follow-up survey (12 weeks after baseline survey) by comparing changes of body mass index (BMI), overweight and obesity prevalence, anthropometric (waist circumference, triceps, subscapular and abdominal skinfold thickness), serum lipids (total cholesterol, high density lipoprotein cholesterol (HDL-C), low density lipoprotein cholesterol (LDL-C), triglycerides), fasting blood glucose and duration of moderate to vigorous physical activity (MVPA) between the intervention and control groups.

## Methods

### Design

The study design was a non-randomized, controlled trail, with cluster sampling, investigating the effectiveness of a school-based PA intervention during 12 weeks from September 2012 to January 2013 on obesity and related health outcomes in school children. Four public schools in Changping District, Beijing of China were selected, all of which were located in the same ethnic, geographic and economic area and purposively selected to ensure similar prevalence of overweight and obesity. After our communications with the head-teachers, two schools of which head-teachers were willing to implement the intervention were allocated to the intervention group, and the other two were allocated to the control group (one elementary school and one middle school in each group). There were two rounds of measurements at baseline survey and follow-up survey (12 weeks after baseline survey). All subjects and measurement staff were blinded to the allocation. The study protocol and data collection instruments were approved by the ethic committee of Peking University Health Science Center. Written informed content was provided by all children and their parents.

### Subjects

Inclusion criteria of subjects included all students of Grade 2 to 5 in elementary schools and Grade 1 to 2 in middle schools, having informed content. By collecting students’ medical history from questionnaires for parents, we excluded the individuals suffering or having a history of any cardiovascular and metabolic diseases, asthma, disabilities. Altogether 921 children aged 7 to 15 years (elementary school students aged 7 to 11, middle school students aged 11 to 15) were enrolled in baseline survey.

### Intervention

Children in the intervention group participated in a multi-component physical activity intervention during 12 weeks that included PE improvement, extracurricular PA for overweight/obese students, PA at home, and health education lectures for students and parents. Children in the control group participated in usual practice.

#### Physical activity components

According to exercise prescription program recommended by American College of Sports Medicine (ACSM) for children and adolescents [33], the PA intervention focused on at least 60-min MVPA per day. The intervention program included three PA components: PE improvement, extracurricular PA for overweight/obese students, and family PA with parent involvement.

Schools were required to improve content, intensity and schedule of PE, to ensure that students have three compulsory 45-minute PE per week, with at least 30-minute MVPA in each class (64%-94% of their age-predicted maximum heart rate [34]; maximum heart rate, calculated as 220-age [33]). For elementary students of Grade 2 and 3, rope jumping and light throwing were mainly practiced; for those of Grade 4 and 5, sprint, endurance running (50 m*8 shuttle run) and rope jumping were practiced; and for middle school students, endurance running (1000 m for boys and 800 m for girls), long jumping and basketball were practiced. During supervision, five students of three physical fitness levels (“good”, “middle” and “poor”) according to recent PE tests were selected to wear a heart rate monitor (Polar RS800CX, Finland). The heart rate curve was exported on computer, so that the intensity of PA and duration of MVPA could be assessed. In the PE without our supervision, PE teachers were required to ask students about self-perceived fatigue levels to judge intensity, and observe their enthusiasm and coordination during the whole class. PE specialists were invited to supervise and guide PE with the field professionals. If the intensity levels were not attained, PE teachers would encourage students to be more active; also, the specialists and professionals would give instructions for course improvement hereafter. Based on supervision with heart rate monitors, students’ feedbacks, PE teachers’ observation and specialists’ suggestion, the field professionals helped PE teachers to achieve the requirement for PE.

Extracurricular PA for overweight and obese students were organized by PE teachers during breaks, at noon, or after school hours, mainly being MVPA such as aerobics, jogging, rope jumping and kinds of games. Though this part was not compulsory, overweight and obese students were encouraged for participation for at least 3 days per week and a total of 30-min MVPA each day were guaranteed. PE teachers encouraged active participation of students, and paid attention to exercise intensity. Like in PE, the teachers were required to ask students about self-perceived fatigue levels and observe their coordination during PA and encourage students to be more active if the intensity was not attained.

The physical activity at home acted as a supplementary part for ensuring 60-min MVPA per day. Students were taught how to judge exercise intensity, and required to write exercise diary each day. If anyone didn't have PE at school, he or she was required to do PA at home for 20 to 30 minutes as a part of homework. We distributed instruction manuals to students, for example, rope jumping for totally 10 minutes (rest time not included) with a 30-second rest after jumping for every minute (primary students) or every two minutes (junior students), jogging for ten minutes (primary students) or fifteen minutes (junior students). Students could choose any kind of PA (not limited to manual content) according to interest and practical condition, but intensity and duration were required to be ensured. Class monitor checked each one's diary every week, and students doing well would get oral praise.

#### Health education lectures

Three health education lectures for students were given by the study team members in each school. The contents of lectures included the cause and harms of childhood obesity, BMI reference for screening overweight and obesity in Chinese school-age children, healthy eating (increasing consumption of vegetables and fruits, reducing consumption of meat, snacks, western fast foods and eating in restaurants, avoiding sugary drinks), and physical activity (intensity, duration, reducing sedentary time). Educational materials were developed by the study team and distributed to the children.

One health education lecture was given to parents or caregivers of children, to briefly introduce knowledge of childhood obesity, the intervention methods and ask for their helps to create household supportive environment for healthy eating and physical activity. They were also required to encourage and supervise their children to have a healthy lifestyle.

### Outcome measures

#### Primary and secondary outcome measures

The primary outcome measures included changes in BMI and overweight/obesity prevalence. The secondary outcome measures included changes in waist circumference, skinfold thickness (triceps, subscapular and abdominal), serum lipids (total cholesterol, HDL-C, LDL-C, and triglycerides), fasting blood glucose and duration of MVPA.

Anthropometric measurements, including height, weight, waist circumference, skinfold thickness were measured according to the standard protocols. Children were asked to wear only light clothes, have bare feet, and stand straight. Weight was measured to the nearest 0.1 kg using a lever scale. Height was measured to the nearest 0.1 cm using a stadiometer. BMI was calculated as body weight (kg) divided by height (m) squared (kg/m^2^). Participants were defined as overweight or obese if the BMI values were higher than the sex- and age-specific cut-off values, according to “BMI Reference for Screening Overweight and Obesity in Chinese School-age Children” developed by Working Group on Obesity in China (WGOC) (Table 1) [35].

**Table 1:** **Body mass index reference for screening overweight and obesity in Chinese school-age children**

Age (years)	Boys		Girls	
	Overweight	Obesity	Overweight	Obesity
7-	17.4	19.2	17.2	18.9
8-	18.1	20.3	18.1	19.9
9-	18.9	21.4	19.0	21.0
10-	19.6	22.5	20.0	22.1
11-	20.3	23.6	21.1	23.3
12-	21.0	24.7	21.9	24.5
13-	21.9	25.7	22.6	25.6
14-	22.6	26.4	23.0	26.3
15-	23.1	26.9	23.4	26.9
16-	23.5	27.4	23.7	27.4
17-	23.8	27.8	23.8	27.7
18	24.0	28.0	24.0	28.0

Waist circumference was measured to the nearest of 0.1 cm, at the navel level. The skinfold thickness on the triceps, subscapularis and abdomen was measured to the nearest of 0.2 mm. The instruments for measurements were calibrated before use. All measurements were conducted by a team of field professionals, who had received standardized training on anthropometric measurements and were blinded to the group allocation.

Fasting venous blood samples were drawn for measurement of total cholesterol, HDL-C, LDL-C, triglycerides, and fasting blood glucose using a biochemical auto-analyzer (Olympus AU400, Japan).

Duration of MVPA was measured by using self-administered questionnaires. This question was designed based on a validated 7-day physical activity questionnaire [36]. Participants were asked to record in-school and out-school MVPA during 7 consecutive days.

#### Process evaluation measures

PE teachers were asked to record PE and extracurricular quantity as well as students’ attendance, self-perceived fatigue levels, enthusiasm and coordination. Every month, PE teachers gave the records to research staff. Research staff also recorded health education lectures quantity, attendance and number of educational materials distributed.

### Statistical analyses

Since the prior data of intraclass correlation (ICC) in Chinese populations is lacking, ICC (0.01) of BMI in a western physical activity intervention [22] was used as reference. The sample size of 921 students from 4 schools had 85% power to detect a mean between-group difference in BMI of 0.33 units.

*Χ*^2^ test and *t* test were used to examine the baseline differences between subjects with complete participation and those lost to follow-up, subjects in intervention group and those in control group with SPSS for Windows 18.0 (SPSS Inc., Chicago, IL, USA).

The primary statistical analyses applied the intention-to-treat principle, i.e. all subjects were analyzed regardless of whether they completed the entire study or not. The baseline values were carried forward imputation for participants missing their final assessment. We used multilevel models to consider cluster random effects and adjust measures at baseline by testing the time × intervention interaction on changes in BMI, waist circumference, skinfold thickness, serum lipids, fasting blood glucose and duration of MVPA (with sex and age as covariates), with MLwiN (version 2.24) software.

For binary data, we implemented a logistic regression model using the same software, with sex and age as covariates. The incidence of overweight/obesity in initially non-overweight/obese students and the remission of overweight/obesity in initially overweight/obese students were analyzed with the same model. We used the 5% significance level.

Additionally we compared the findings of intention-to-treat analyses with that of analyses using data of subjects who completed the entire study.

## Results

### General characteristics of subjects

There were 921 students (489 boys and 432 girls) recruited at baseline survey in September 2012, with 388 (204 boys and 184 girls) and 533 (285 boys and 248 girls) students in intervention group and control group respectively. No significant difference of sex ratio and duration of MVPA were observed between two groups (all *P* > 0.05). Participants had mean age of 10.4 years, mean BMI of 19.59 kg/m^2^. Students in intervention group were slightly younger (*P* = 0.009) and had smaller BMI (*P* = 0.006) than those in control group. There were 339 (36.8%) overweight or obese students in all subjects. The prevalence of overweight/obesity in intervention group was smaller than that of control group (*P* = 0.040). Except for LDL-C, the differences of waist circumference, skinfold thickness, serum lipids and fasting glucose between two groups were significant (all *P* < 0.050) (Table 2).

**Table 2:** **General characteristics of subjects at baseline**

Characteristic	Total	Intervention group	Control group	***P***value
	(N = 921)	(N = 388)	(N = 533)	
Age, mean (SD), y	10.4 (2.2)	10.2 (2.3)	10.6 (2.2)	0.009
Sex, no. (%)				
Boys	489 (53.1)	204 (52.6)	285 (53.5)	0.788
Girls	432 (46.9)	184 (47.4)	248 (46.5)	
Body mass index, mean (SD), kg/m^2^	19.59 (4.41)	19.12 (4.28)	19.93 (4.47)	0.006
Overweight/obesity, no. (%)	339 (36.8)	128 (33.0)	211 (39.6)	0.040
Waist circumference, mean (SD), cm^a^	67.94 (12.74)	65.68 (12.62)	69.58 (12.58)	<0.001
Skinfold thickness, mean (SD), mm^a^				
Triceps skinfold	15.08 (7.03)	14.45 (7.28)	15.55 (6.82)	0.019
Subscapular skinfold	12.75 (7.86)	11.94 (8.13)	13.34 (7.61)	0.007
Abdominal skinfold	17.46 (11.80)	15.84 (12.34)	18.64 (11.26)	<0.001
Serum lipids, mean (SD), mmol/L^a^				
Total cholesterol	3.94 (0.68)	4.02 (0.68)	3.88 (0.68)	0.002
Triglycerides	0.81 (0.41)	0.85 (0.40)	0.79 (0.42)	0.031
HDL-C	1.43 (0.28)	1.46 (0.29)	1.40 (0.28)	0.004
LDL-C	2.38 (0.60)	2.34 (0.58)	2.41 (0.62)	0.088
Fasting glucose, mean (SD), mmol/L^a^	4.79 (0.49)	4.99 (0.46)	4.65 (0.45)	<0.001
Duration of MVPA (min/day)^b^	45.8 (55.1)	44.7 (45.7)	46.6 (60.7)	0.627
In school	29.0 (35.2)	27.4 (26.0)	30.1 (40.3)	0.283
Out of school	16.8 (30.1)	17.2 (28.6)	16.4 (31.1)	0.716

^a^Denominators varied because of missing data. (In intervention group, 2 missed in waist circumference, 4 missed in serum lipids and fasting glucose; in control group, 1 missed in waist circumference and skinfold thickness, 3 missed in serum lipids and fasting glucose).

^b^Subjects with complete 7-day records were included in analysis (334 in intervention group, 483 in control group).

HDL-C: high density lipoprotein cholesterol.

LDL-C: low density lipoprotein cholesterol.

MVPA: moderate to vigorous physical activity.

**Figure 1:**
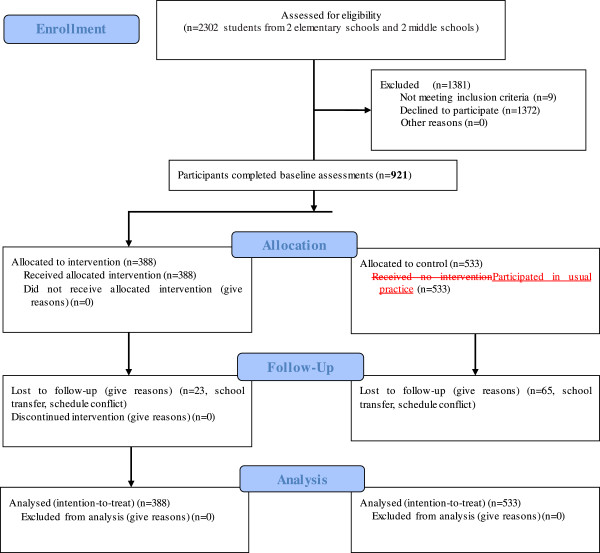
**Flow diagram of the study.**

After 12 weeks, 833 (90.4%) children accepted the follow-up survey, while 88 students (23 in intervention group and 65 in control group) had no follow-up data because of school transfer, schedule conflict (Figure 1). The retention rate was 94.1% in intervention group and 87.8% in the control group. No significant differences in age, sex, duration of MVPA, BMI and overweight/obesity prevalence were observed between completers and those lost to follow-up survey (all *P* > 0.050) (Table 3).

**Table 3:** **Comparison of basic characteristics between subjects lost to follow-up and those with complete participation at baseline**

Characteristic	Subjects lost to follow-up	Subjects with complete participation	***P***value
	(N = 88)	(N = 832)	
Age, mean (SD), y	10.5 (1.9)	11.0 (2.3)	0.067
Sex, no. (%)			
Boys	43 (48.9)	446 (53.5)	0.403
Girls	45 (51.1)	387 (46.5)	
Body mass index, mean (SD), kg/m^2^	18.78 (4.33)	19.67 (4.41)	0.070
Overweight/obesity, no. (%)	31 (35.2)	308 (37.0)	0.746
Duration of MVPA (min/day)^a^	37.1 (35.0)	46.7 (56.7)	0.141
In school	25.7 (24.3)	29.4 (36.1)	0.369
Out of school	11.5 (17.7)	17.3 (31.1)	0.100

^a^Subjects with complete 7-day records were included in analysis (88 subjects lost to follow-up, 738 subjects with complete participation).

MVPA: moderate to vigorous physical activity.

### Primary outcomes

Table 4 showed the results of intention-to-treat analyses for the primary outcomes, as well as the adjusted OR and difference. The reduction of BMI was statistically significant in intervention group (−0.02 ± 0.06 kg/m^2^), compared with increase of BMI in control group (0.41 ± 0.08 kg/m^2^; adjusted mean difference, −0.43 kg/m^2^; 95% CI, −0.63 to −0.23 kg/m^2^; *P* < 0.001). The overweight/obesity prevalence decreased by 2.3% after 12 weeks in intervention group, compared with the increase in control group (1.7%), but the difference was not significant (*P* = 0.370).

**Table 4:** **Changes in BMI and overweight/obesity prevalence in intervention and control groups**

	Intervention group	Control group
	(N = 388)	(N = 533)
Body mass index, kg/m^2^		
Baseline, mean (SD)	19.12 (4.28)	19.93 (4.47)
Follow-up, mean (SD)	19.10 (4.08)	20.33 (4.59)
Mean change (SE)	−0.02 (0.06)	0.41 (0.08)
Adjusted difference, mean (95% CI)^a^	−0.43 (−0.63 to −0.23)	
*P* value	<0.001	
Overweight/obesity		
Baseline, %	33.0	39.6
Follow-up, %	30.7	41.3
Change, %	−2.3	1.7
Change difference, %	−4.0	
Adjusted OR (95% CI)^bc^	0.84 (0.56 to 1.24)	
*P* value	0.370	

^a^Adjusted difference was calculated using multilevel model adjusted for sex and age.

^b^Adjusted odds ratio was calculated using logistic regression model adjusted for sex and age.

^c^OR: The risk of becoming overweight or obesity in the intervention group was 0.84 times more than children in the control group, showing a reduction of 16.0% the risk of being overweight or obese.

**Figure 2:**
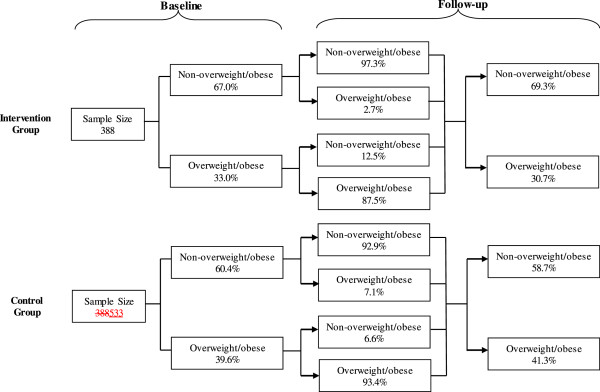
**Changes in proportion of subjects’ nutritional status from baseline to follow-up survey in intervention and control group.**

The incidence of overweight/obesity in intervention group was significantly lower than that in control group (2.7% vs. 7.1%, adjusted OR (95% CI), 0.34(0.15 to 0.80); *P* = 0.015). Remission of overweight/obesity in intervention group was higher than that in control group, but the difference was not significant (12.5% vs. 6.6%, *P* = 0.370) (Figure 2).

### Secondary outcomes

Table 5 showed the results of intention-to-treat analyses for secondary outcomes. The mean changes of triceps, subscapular and abdominal skinfold thickness were all significantly different between the intervention and control groups (adjusted mean difference (95% CI): −1.40 mm (−1.87 to −0.93) mm, *P* < 0.001; −1.26 mm (−1.84 to −0.67) mm, *P* < 0.001; −0.69 mm (−1.29 to −0.10) mm, *P* = 0.023).

**Table 5:** **Changes in anthropometric and metabolic indicators and MVPA duration in intervention and control groups**

Measure		Intervention group				Control group			Adjusted difference, mean (95% CI) ^a^	***P***value
	N	Baseline mean (SD)	Follow-up mean (SD)	Mean (SE) change	N	Baseline mean (SD)	Follow-up mean (SD)	Mean (SE) change		
Waist circumference, cm	386	65.68 (12.62)	64.61 (11.69)	−1.06 (0.17)	532	69.58 (12.58)	68.84 (12.25)	−0.70 (0.14)	−0.38 (−0.81 to 0.05)	0.393
Skinfold thickness, mm										
Triceps skinfold	388	14.45 (7.28)	14.21 (7.06)	−0.24 (0.18)	532	15.55 (6.82)	16.68 (7.89)	1.15 (0.16)	−1.40 (−1.87 to −0.93)	<0.001
Subscapular skinfold	388	11.94 (8.13)	11.77 (7.89)	−0.18 (0.23)	532	13.34 (7.61)	14.40 (9.55)	1.08 (0.19)	−1.26 (−1.84 to −0.67)	<0.001
Abdominal skinfold	388	15.84 (12.34)	14.64 (11.80)	−1.22 (0.22)	532	18.64 (11.26)	18.09 (12.36)	−0.54 (0.20)	−0.69 (−1.29 to −0.10)	0.023
Serum lipids, mmol/L										
Total cholesterol	384	4.02 (0.68)	3.90 (0.65)	−0.12 (0.02)	530	3.88 (0.68)	3.80 (0.64)	−0.08 (0.02)	−0.05 (−0.11 to 0.02)	0.143
Triglycerides	384	0.85 (0.40)	0.93 (0.42)	0.09 (0.02)	530	0.79 (0.42)	0.88 (0.46)	0.10 (0.02)	−0.01 (−0.06 to 0.03)	0.599
HDL-C	384	1.46 (0.29)	1.44 (0.30)	−0.02 (0.01)	530	1.40 (0.28)	1.36 (0.28)	−0.04 (0.01)	0.02 (−0.003 to 0.05)	0.085
LDL-C	384	2.34 (0.58)	2.28 (0.54)	−0.07 (0.02)	530	2.41 (0.62)	2.34 (0.56)	−0.07 (0.02)	0.003 (−0.04 to 0.05)	0.910
Fasting glucose, mmol/L	384	4.99 (0.46)	4.97 (0.44)	−0.03 (0.02)	530	4.65 (0.45)	4.81 (0.41)	0.17 (0.02)	−0.19 (−0.24 to −0.15)	<0.001
Duration of MVPA, min/day	334	44.7 (45.7)	53.5 (66.2)	8.9 (4.3)	483	46.6 (60.7)	32.8 (47.3)	−13.8 (3.3)	22.7 (12.2 to 33.2)	<0.001
In school	334	27.4 (26.0)	34.3 (46.1)	6.8 (2.9)	483	30.1 (40.3)	18.9 (28.3)	−11.2 (2.1)	18.1 (11.2 to 25.0)	<0.001
Out of school	334	17.2 (28.6)	19.3 (35.0)	2.0 (2.4)	483	16.4 (31.1)	13.9 (26.9)	−2.6 (1.8)	4.6 (−1.0 to 10.2)	0.117

^a^Adjusted differencewas calculated using multilevel models adjusted for sex and age.

HDL-C: high density lipoprotein cholesterol. LDL-C: low density lipoprotein cholesterol. MVPA: moderate to vigorous physical activity.

The mean change of fasting glucose was significantly different in intervention group (−0.03 ± 0.02 mmol/L), compared with that in control group (0.17 ± 0.02 mmol/L; adjusted mean difference, −0.19 mmol/L; 95% CI, −0.24 to −0.15 mmol/L; *P* < 0.001). There was no significant difference in waist circumference and serum lipids levels between two groups (all *P* > 0.050).

The increase of in-school MVPA duration was statistically significant in intervention group (6.8 ± 2.9 min/day), compared with reduction of that in control group (−11.2 ± 2.1 min/day; adjusted mean difference, 18.1 min/day; 95% CI, 11.2 to 25.0 min/day; *P* < 0.001). Although not significant, out-school MVPA duration increased by 2.0 ± 2.4 min/day in intervention group, while decreased by 2.6 ± 1.8 min/day in control group. The increase of total MVPA duration was statistically significant in intervention group (8.9 ± 4.3 min/day), compared with reduction of that in control group (−13.8 ± 3.3 min/day; adjusted mean difference, 22.7 min/day; 95% CI, 12.2 to 33.2 min/day; *P* < 0.001).

We additionally analyzed data of completers and found the results were in line with the above intention-to-treat analyses for both primary and secondary outcomes (data not shown).

### Process evaluation

All 388 participants in intervention group took part in PE. PE was guaranteed 3 times per week in both schools. In 720 PE of two schools, the attendance rate reached 95%-100%; 82.7% reached at least moderate PA level; 79.5% and 85.3% had students’ high enthusiasm and high coordination respectively. Among 128 overweight and obese students who were encouraged to take part in extracurricular PA, 100% had participation of 3 times per week. In 60 extracurricular PA, 87.5% reached at least moderate PA level.

Each intervention school had three health education lectures for students, each lasting for 30 to 40 minutes, with attendance rate of 95%-100%, and one health education lecture for parents, lasting 25-30minutes, with attendance rate of 100%. Distribution rate of educational materials was 100%.

### Adverse events

The intervention did not increase the number of underweight children in the intervention group (11 and 7 underweight children before and after the intervention, respectively). Based on the questionnaire data, this intervention did not increase body dissatisfaction rate (16.0% and 4.7% in intervention group vs. 25.5% and 22.9% in control group before and after the intervention, respectively; *P* = 0.411). Wearing heart rate monitor posed little harm to participants. PE and extracurricular PA were organized by professional PE teachers, and PA at home was supervised by parents to guarantee safety. We asked the students to report any physical discomfort or injures during PA. No other adverse events were reported.

## Discussion

The non-randomized controlled trial showed that a school-based physical activity intervention in 7 to 15-year-old children for 12 weeks significantly decreased levels of BMI, skinfold thickness and fasting glucose, and increased duration of MVPA.

Compared with previous studies in Chinese children with simple school-based PA intervention methods, our intervention had multi-component PA intervention with the following features. Firstly, it is the first study in China that improved intensity and duration of PE. PE is an important component in school PA, because it covers all students and the attendance could be ensured. However, the phenomenon of not providing PE or replacing PE with other indoor class is widespread in Chinese schools [37]. So the present study tried to improve PE, making the best use of the available PA time. Secondly, the ACSM suggested that the duration of PA for the purpose of weight loss should be longer than that for health maintenance, and the intensity be at least moderate [33]. Overweight and obese children were specially organized for extracurricular PA, which can guarantee duration and intensity under supervision of professional PE teachers in schools. Thirdly, reviews have indicated that physical activity interventions among young people involving family enhanced the effectiveness of interventions delivered in the school setting [29, 38]. This intervention included PA at home as a supplementary part for ensuring 60-min MVPA per day, having parent involvement as the supportive environment. Finally, it was suggested that changes of PA behaviors plus healthy eating would have significant long-term weight loss effect [39]. Therefore, we added health education lectures as a component of intervention to encourage children to have not only active lifestyle but also healthy eating.

Prevalence of childhood obesity as an indicator of public health problems should be paid attention, but there were few studies of obesity interventions that focused on this in children [26]. Although the difference of overweight/obesity prevalence was not significant between two groups, our study resulted in a positive trend (change difference −4.0%). Meta-analysis showed that the longer the intervention period, the greater the decrease in likelihood of being overweight and obese compared to shorter duration intervention period [40]. We strongly expected a significant prevalence change in the future considering the positive results of MVPA and BMI of the intervention. The incidence of overweight/obesity in intervention group was significantly lower than that in controls, which was in line with another PA intervention [41]. It indicated that this intervention could have favorable impact on children at risk of becoming obese. The remission of overweight/obesity in the intervention group was higher than that in control group, but the difference was not significant. It may be due to the shorter interventional duration, compared to another 3-year study with statistically significant result of obesity remission [26]. The intervention also led to significant between-group difference in BMI change (adjusted mean difference, −0.43 kg/m^2^), and changes of triceps, subscapular and abdominal skinfold thickness. The above positive changes did not increase the number of underweight children. In recent well-designed reviews, effects of lifestyle or PA interventional on obesity prevalence and BMI were equivocal [25, 40]. Establishing a programme with consistent positive effects on body composition is of public health importance [22]. The positive effect on body composition in our study is important for public health, as a higher childhood BMI has been associated with coronary heart disease in adulthood [42], a higher BMI in adolescence predicted adverse health effects in adults even without obesity in adulthood [43], and childhood obesity will become a high economic and social burden for society [44].

This intervention led to an increase in total MVPA per day, which was consistent with another school-based PA intervention [45]. The significant increase of in-school MVPA proved school’s attractive points as targeted setting once again. There was no significant difference in serum lipids levels between two groups. Studies with significantly improved blood lipids usually had interventional duration longer than 4 months [46, 47]. It suggested that obesity reduction may need more time to improve cardiovascular risk factors. Our intervention resulted in significant fasting glucose change of −0.19 mmol/L, which was in line with an 18-week school-based PA programme [48]. It has been demonstrated that PA could alter insulin sensitivity independent of changes in weight and body composition in children [19, 49]. So PA is important for not only obesity reduction, but also fasting glucose improvements in children. More precise indicators of insulin sensitivity are needed in further validation studies.

PE that our program targeted and break-time that we used for extracurricular PA are compulsory in Chinese schools and are guaranteed by a new policy released by the Department of Education in 2011 [50]. We just improved the quality without making extra burden to schools, and we got good feedback from teachers and students. As for family PA, we not only provided guidance, but also asked parents to encourage and supervise their children at home. Concerning health education, the materials had been distributed to participants. And schools could invite experts for lectures if they are willing to. Under the above consideration, we predicted the intervention effects would sustain although it only lasted for 12 weeks. However, further research is needed to identify its long-term effects.

### Generalizability and transferability

Although differences exist between school systems in different countries, our program has generalizability to some extent. Firstly, global epidemic of childhood obesity [3] has raised public concern and attention, which may promote support for implementation. Secondly, worldwide, PE is by far the most common method of promoting PA in school days and most countries have legal requirements for school PE for at least some parts of compulsory school years; even in countries where PE may not be mandated by law, it is still offered [51]. In China, PE quantity per week and PA skills to teach are required according to different grades, and MVPA should account for 25 (40-min class) to 30 (45-min class) minutes per class with students’ interests raised. Besides, the training for PE teachers also constitutes effective and quality teaching in PE in China. Thirdly, break-time in school days provides valuable opportunities for young people to participate in organized PA [51]. What we have to do is to make the best use of it for overweight and obese students. In counties where it may be less acceptable to specifically target overweight or obese children, we suggest organizing break-time PA for all children, since inclusion of all children avoids stigmatization of overweight or obese children and gives all children an equal chance to benefit from the intervention. Besides, overweight or obese children are encouraged to have more out-school PA, such as riding or walking. Finally, family or parent involvement is not rare in childhood obesity intervention internationally [24]. What our program emphasizes is providing family PA guidance and promoting parents’ encouragement and supervision for their children’s healthy lifestyles. Considering the lack of parental involvement still exists in some interventions, there is a need to further explore cultural barriers as to why this might be the case [52].

### Strengths and limitations

This study contributes an effective and successful way of implementing a PA programme in school setting to reduce childhood obesity and improve obesity related health outcomes. The results showed that a school-based multi-component PA intervention including improvement of PE, extracurricular PA for overweight/obese children, PA at home, and health education lectures for students and parents can decrease levels of BMI, skinfold thickness and fasting glucose. The experience of intervention can provide important evidence for controlling increase of childhood obesity prevalence in countries with childhood obesity trend and school settings similar to China and for further research on childhood obesity intervention. Moreover, the PA interventional methods in this study were based on guidelines recommended by ACSM and previous Chinese studies. Additionally, instead of focusing on in-school PA in previous studies [27], the intervention also included PA at home to guarantee 60-min MVPA each day and parent involvement to create supportive environment. This may explain the success of the study in relatively shorter interventional duration.

Due to practical reasons, our study was a non-randomized controlled trial, which is a limitation of the study. It resulted in incomparable baseline data between the two groups. However, school consent to run the intervention program within the school curriculum was necessary prior to commencement of the study [53]. We used the statistical methods to compensate for the deficiency to some extent. In addition, our intervention duration was relatively short, which could not detect significant changes of serum lipids. The study only detected short-term intervention effects, but the long-term effects were uncertain. A review indicated that school-based interventions of 12 to 16 weeks work well from a practical point of view [54]. So the influence of interventional duration on effectiveness should be further explored.

## Conclusion

The school-based, multi-component physical activity intervention significantly decreased levels of BMI, skinfold thickness and fasting glucose, and increased duration of MVPA. These findings suggest that implementation of such intervention would help to reduce adiposity and improve health and provided evidence for the development of effective and feasible school-based obesity interventions.
